# Exogenous glucosamine globally protects chondrocytes from the arthritogenic effects of IL-1β

**DOI:** 10.1186/ar2082

**Published:** 2006-11-16

**Authors:** Jean-Noël Gouze, Elvire Gouze, Mick P Popp, Marsha L Bush, Emil A Dacanay, Jesse D Kay, Padraic P Levings, Kunal R Patel, Jeet-Paul S Saran, Rachael S Watson, Steven C Ghivizzani

**Affiliations:** 1 Department of Orthopaedics and Rehabilitation, Gene Therapy Laboratory, University of Florida, College of Medicine, PO Box 100137, Gainesville, FL 32610-0137, USA; 2 Interdisciplinary Center for Biotechnology Research, University of Florida, Gainesville, FL 32610-0137, USA

## Abstract

The effects of exogenous glucosamine on the biology of articular chondrocytes were determined by examining global transcription patterns under normal culture conditions and following challenge with IL-1β. Chondrocytes isolated from the cartilage of rats were cultured in several flasks either alone or in the presence of 20 mM glucosamine. Six hours later, one-half of the cultures of each group were challenged with 10 ng/ml IL-1β. Fourteen hours after this challenge, RNA was extracted from each culture individually and used to probe microarray chips corresponding to the entire rat genome. Glucosamine alone had no observable stimulatory effect on the transcription of primary cartilage matrix genes, such as aggrecan, collagen type II, or genes involved in glycosaminoglycan synthesis; however, glucosamine proved to be a potent, broad-spectrum inhibitor of IL-1β. Of the 2,813 genes whose transcription was altered by IL-1β stimulation (*P *< 0.0001), glucosamine significantly blocked the response in 2,055 (~73%). Glucosamine fully protected the chondrocytes from IL-1-induced expression of inflammatory cytokines, chemokines, and growth factors as well as proteins involved in prostaglandin E_2 _and nitric oxide synthesis. It also blocked the IL-1-induced expression of matrix-specific proteases such as MMP-3, MMP-9, MMP-10, MMP-12, and ADAMTS-1. The concentrations of IL-1 and glucosamine used in these assays were supraphysiological and were not representative of the arthritic joint following oral consumption of glucosamine. They suggest, however, that the potential benefit of glucosamine in osteoarthritis is not related to cartilage matrix biosynthesis, but is more probably related to its ability to globally inhibit the deleterious effects of IL-1β signaling. These results suggest that glucosamine, if administered effectively, may indeed have anti-arthritic properties, but primarily as an anti-inflammatory agent.

## Introduction

Osteoarthritis (OA) is a chronic, disabling condition for which there is no cure and few useful treatments. OA primarily affect the hips, knees and distal interphalangeal joints of the hands and is generally associated with a progressive loss of articular cartilage accompanied by sclerosis of the subchondral bone [1,2]. Clinical features include joint pain, instability, limitation of motion and functional impairment. The pathogenesis of OA, although not yet well understood, is often linked to joint injury, biomechanical alterations and aging. Many investigators consider cytokines, such as IL-1, as well other inflammatory mediators synthesized locally by synovial cells and chondrocytes, to be key contributors to the progression of the disease [3,4].

The failure of conventional pharmacologics to satisfactorily control OA probably explains the increasing use of self-treatments such as glucosamine and other 'nutraceuticals' [5-7]. Indeed, over the past several years, glucosamine has been widely endorsed by the lay-press as a useful over-the-counter remedy for OA, with estimated annual sales exceeding $700 million in the United States alone.

Although anecdotal evidence of the capacity of glucosamine to relieve OA symptoms is widespread, its mode of action is ill-defined. D-Glucosamine, the biologically active form, serves as a metabolic precursor in the synthesis of several classes of compounds requiring amino sugars, including the proteoglycans, glycosaminoglycans, and hyaluronate. Because these compounds are essential extracellular matrix (ECM) components of connective tissues, a common perception is that oral consumption of large quantities of glucosamine leads to elevated intra-articular concentrations and thereby enhances synthesis of the articular cartilage matrix. This belief, however, has never been conclusively demonstrated *in vivo*. Reports of the efficacy of glucosamine have been inconsistent in controlled clinical studies, leaving doubts among the scientific community and skepticism that its ingestion as a dietary supplement mediates a meaningful biological response in the joint tissues [8-11]. Indeed, the recent findings of the multicenter, double-blind, placebo-controlled Glucosamine/chondroitin Arthritis Intervention Trial were somewhat mixed [12]. This trial, intended to resolve and clarify the clinical effectiveness of these supplements with regard to OA, has perhaps had the reverse effect and has fueled the controversy.

In attempts to describe more clearly the effects of elevated glucosamine on cartilage biology, several laboratory studies have been undertaken that suggest glucosamine may have specific chondroprotective properties. Initial work *in vitro *showed that glucosamine could moderate certain aspects of the deleterious response of chondrocytes to stimulation with IL-1 [13] or lipopolysaccharide [14]. These aspects included inhibition of phospholipase A_2 _activity [15], prostaglandin E_2 _and nitric oxide (NO) synthesis [13], reduced COX-2 mRNA and protein expression [16,17], and protection from reduced proteoglycan synthesis in articular cartilage [13,18-20]. Inhibition of aggrecanase-dependent cleavage of aggrecan was also observed in both rat and bovine cartilage explant cultures when supplemented with glucosamine [21]. In addition, NF-κB activation as well as the nuclear translocation of p50 and p65 proteins was inhibited in chondrocytes cultured in the presence of glucosamine, suggesting that glucosamine may block inflammatory signaling [17,22].

Studies such as those already cited involving assays of individual genes and proteins have provided only a limited indication of the response of articular chondrocytes to elevated levels of exogenous glucosamine. Given the popularity of glucosamine as a means to manage OA symptoms, and discrepancies regarding its possible mode of action and true value as an anti-arthritic, we performed gene expression analyses using microarrays in an effort to determine how elevated levels of exogenous glucosamine influence the global gene expression patterns of articular chondrocytes. We found that addition of glucosamine to the culture medium had no apparent stimulatory effect on the expression of biosynthetic genes but was a surprisingly effective inhibitor of IL-1β, blocking its effects on thousands of genes.

## Materials and methods

### Chondrocyte isolation and culture

Articular cartilage was isolated from the femoral heads of male Wistar rats under aseptic conditions (Charles River Laboratories, Boston, MA, USA). Chondrocytes were obtained by sequential digestion of the cartilage with pronase and type II collagenase (Invitrogen, Carlsbad, CA, USA) as previously described [23]. After filtration to remove tissue debris, the cells were cultured in 75-cm^2 ^flasks in complete DMEM (supplemented with 10% fetal bovine serum and 1% penicillin–streptomycin; Invitrogen) at 37°C in a humidified atmosphere containing 5% CO_2_.

Experiments were subsequently performed with second-passage cultures, whereby the cells from the large cultures were trypsinized, pooled and seeded into 20 flasks of 25 cm^2 ^volume. These flasks were then divided into four treatment groups to evaluate the effects of glucosamine and IL-1 on global transcription patterns (*n *= 5/group). To the culture medium in one-half of the flasks was added glucosamine and HCl (Sigma-Aldrich, St Louis, MO, USA) to a final concentration of 20 mM [13]. Six hours later, IL-1β was added at 10 ng/ml to five of the flasks receiving glucosamine and to five of the untreated flasks. Fourteen hours post IL-1β stimulation, and immediately prior to RNA isolation, the conditioned media were collected from all cultures and analyzed individually for NO production as indicated by the nitrite levels. The total RNA was then isolated individually from the respective cultures.

### Nitrite assay

NO production was determined spectrophotometrically by measuring in conditioned medium the accumulation of nitrite (NO_2_^-^), a stable breakdown product of NO. Nitrate in the media were first converted to nitrite by the action of nitrate reductase from *Aspergillus niger *(Roche, Florence, SC, USA). Then 100 μl culture supernatant was mixed with 100 μl Griess reagent (sulfanilamide (1% w/v)) in 2.5% H_3_PO_4 _and *N*-naphthylethylenediamine dihydrochoride ((0.1% w/v) in H_2_O), and was incubated at room temperature for 5 min in 96-well plates. The absorbance at 550 nm was measured on a Multiskan MCC microplate reader (Thermo, Waltham, MA, USA). The nitrite concentration was calculated from a standard curve of sodium nitrite and expressed as the micromolar concentration [24].

After comparison of data by analysis of variance the different groups were compared using Fisher's *t *test. Assays were performed in quintuplet. *P *< 0.05 was considered significant.

### Preparation of labeled copy RNA

The total RNA from each chondrocyte culture was extracted individually and prepared for hybridization according to the GeneChip Expression Analysis Technical Manual (2001; Affymetrix, Santa Clara, CA, USA). Briefly, cells were lysed in the presence of Trizol solution (Sigma-Aldrich, St Louis, MO, USA). Following extraction of the homogenate with chloroform, the total RNA was precipitated with isopropanol and resuspended in 10 mM Tris–HCl, pH 8.0, 1 mM ethylenediamine tetraacetic acid. Newly extracted RNA was then cleaned using RNeasy mini columns as described by the manufacturer (RNeasy Mini Protocol for RNA cleanup; Qiagen, Valencia, CA USA).

The amount and quality of each RNA sample were assessed by spectrophotometry. The four samples from each treatment with the greatest OD 260/280 ratios were used for target labeling as follows. A 3 μg aliquot of total RNA was used as a template for cDNA synthesis (One-Cycle cDNA Synthesis Kit; Affymetrix). First-strand synthesis and second-strand synthesis were performed following the manufacturer's instructions. The second-strand product was cleaned (GeneChip Sample Cleanup Module; Affymetrix) and used as a template for *in vitro *transcription with biotin-labeled ribonucleotides (GeneChip IVT Labeling Kit; Affymetrix). The resulting cRNA product was cleaned (GeneChip Sample Cleanup Module; Affymetrix), and a 20-μg aliquot was heated at 94°C for 35 minutes in the fragmentation buffer provided with the cleanup module (Affymetrix).

### Array hybridization

Microarray hybridization and data analyses were performed by the Gene Expression Core of the Interdisciplinary Center for Biotechnology Research at the University of Florida. Fifteen micrograms of adjusted cRNA from each sample was hybridized for 16 hours at 45°C to Affymetrix GeneChip Rat Genome 230 2.0 arrays (Affymetrix). After hybridization, each chip was stained with a streptavidin–phycoerythryn conjugate (Invitrogen-Molecular Probes, Carlsbad, CA, USA), was washed, and was visualized with a microarray scanner (Genearray Scanner; Agilent Technologies, Santa Clara, CA, USA). Images were inspected visually for hybridization artifacts. In addition, quality assessment metrics were generated for each scanned image and were evaluated based on empiric data from previous hybridizations and on the signal intensity of internal standards that were present in the hybridization cocktail. Samples that did not pass quality assessment were eliminated from analyses.

### Generation of expression values

Microarray Suite (version 5; Affymetrix) was used to generate *.cel files, and a computer program (Probe Profiler, version 1.3.11; Corimbia, Inc Berkeley, CA, USA) developed specifically for the GeneChip system (Affymetrix) was used to convert intensity data into quantitative estimates, globally scaled to 100, of gene expression for each probe set. The software identifies informative probe pairs and downweights the signal contribution of probe pairs that are subject to differential cross-hybridization effects or that consistently produce no signal. The software also detects and corrects for saturation artifacts, outliers, and chip defects. A probability statistic was generated for each probe set. The probability is associated with the null hypothesis that the expression level of the probe set is equal to 0 (background). Genes not significantly expressed above the background in any of the samples (*P *< 0.05) were considered absent and removed from the data set.

### Data analysis

A one-way analysis of variance for replicates was performed on expression values to evaluate the presence of a treatment effect (*P *< 0.0001). Genes for which there was a significant treatment effect were subjected to a Tukey's honest significant difference post-hoc test (*P *< 0.05). The expression values of those genes considered to have a significant effect were normalized by performing a Z-transformation, thereby generating a distribution with mean 0 and standard deviation 1 for each gene. K-means clustering and principal component analysis were performed on normalized values (GeneLinker Gold 3.1, Kingston, ON, Canada).

### Real-time PCR analysis

cDNA was synthesized from 1 μg total RNA using M-MLV reverse transcriptase and was primed with random hexamer oligonucleotides (Invitrogen) in a 20 μl reaction. Amplification by PCR was carried out in a 25 μl reaction volume using a SYBR Green MasterMix (Eppendorf, Hamburg, Germany). Relative expression levels were normalized to EF1α and calculated using the 2^-Δ*Ct *^method [25]. Primer sequences for the genes of interest are presented in Table 1. After comparison of the data by analysis of variance, the different groups were compared using Fisher's *t *test (*n *= 3; *P *< 0.05 considered significant).

The data discussed in this publication have been deposited in the National Center for Biotechnology Information Gene Expression Omnibus [26] and are accessible through Gene Expression Omnibus Series accession number GSE6119.

## Results

In an effort to describe more fully the influence of glucosamine on the metabolism of articular chondrocytes, we studied the effects of exogenous glucosamine and IL-1β on global expression patterns using microarrays. Articular chondrocytes from rats were seeded into several flasks, and the media in one-half was supplemented with glucosamine at 20 mM. Six hours later, IL-1β at 10 ng/ml was added to one-half of the flasks receiving glucosamine and to one-half of the untreated flasks. Fourteen hours post IL-1β stimulation, the conditioned media were collected and analyzed for NO production.

**Figure 1:**
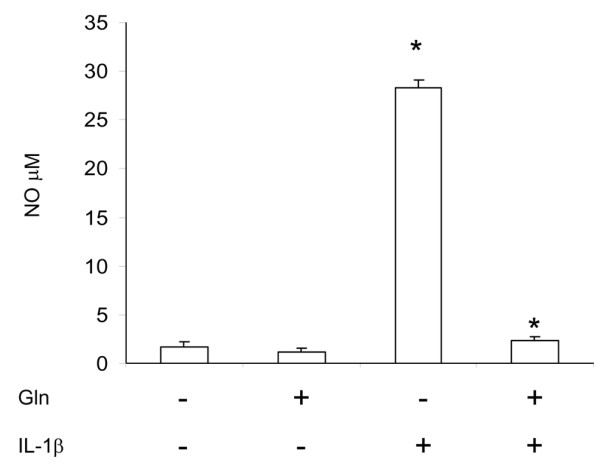
Nitric oxide production in chondrocytes following culture with elevated glucosamine and subsequent challenge by IL-1β. Articular chondrocytes from rats were seeded into 20 flasks, which were divided into four groups. Glucosamine was added to a final concentration of 20 mM to two of the groups. Six hours later, 10 ng/ml IL-1β was added to one group receiving glucosamine and to one previously untreated group. NO production was assessed by measurement of nitrite in the conditioned media from the four respective groups: untreated control, glucosamine (Gln) alone, IL-1β alone, and glucosamine with IL-1β. Results are expressed in μM nitrite, each bar representing the mean of five assays. Error bars represent one standard deviation. *P < 0.05 versus glucose control.

Previous studies have shown that, under appropriate conditions, glucosamine is an effective inhibitor of IL-1β-induced NO synthesis in chondrocytes. To help ensure that subsequent microarray data provided an accurate representation of the effects of glucosamine and IL-1β on chondrocyte transcription, NO levels were used to verify that the cultures were viable and responded fully and reproducibly to both molecules [13,22]. As shown in Figure 1, IL-1β alone was a potent stimulus for NO production, generating a >10-fold increase in conditioned media over background levels (28.3 ± 0.8 μM versus 2.4 ± 0.4 μM, respectively). In cultures receiving glucosamine and IL-1β, NO synthesis was essentially at background levels. Having confirmed that the culture systems were functioning optimally, total RNA was extracted separately from each flask and the OD 260/280 ratios were determined. RNA samples with ratios greater than 1.8 were used to prepare labeled cRNA probes, which were then hybridized to individual Affymetrix 230 2.0 array chips representing the complete rat genome.

**Figure 2:**
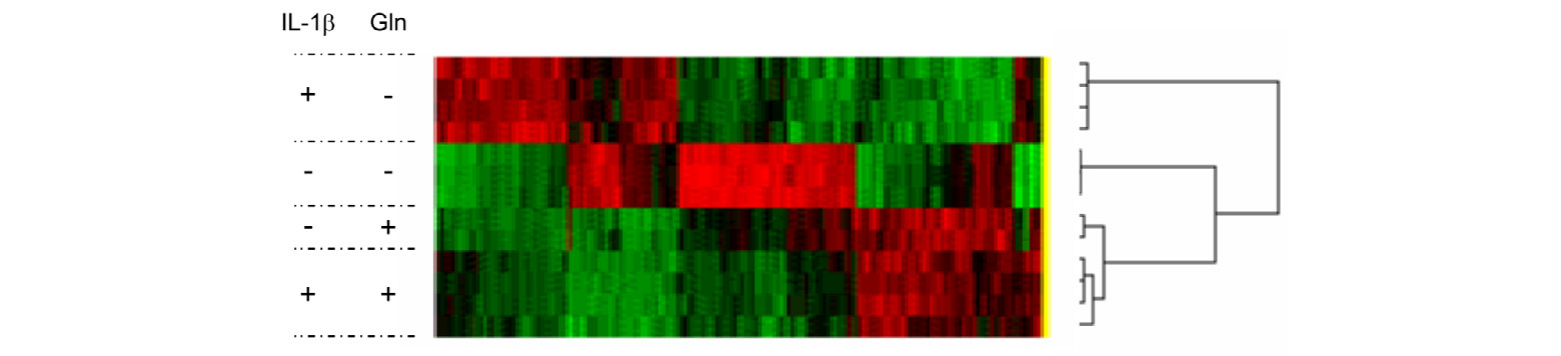
Changes in global expression patterns of chondrocytes induced by glucosamine and IL-1β. A two-way agglomerative hierarchical clustering of samples. The treatment groups are indicated by the legend on the left (also see text; Gln, glucosamine). Only the normalized signal values of genes with a significant (*P *< 0.0001) treatment effect were included. Each row represents a sample and each column a gene. Color intensities reflect relative signal values, whereby red represents a higher level of gene expression, and green a lower level relative to the mean across all samples for each gene. On the right, hierarchical clustering of the samples is indicated both within and among treatment groups. Longer lines represent greater dissimilarity between samples. For these samples, one of the untreated control samples and two samples from the glucosamine-alone groups were eliminated from the final analysis because they did not satisfy the quality control criteria of the microarray analysis.

Of the 31,042 probe sets (genes) present on the array, 27,061 were detected significantly above background on at least one array set. Analysis of the overall results of the hybridization using hierarchical clustering of the samples showed a high degree of similarity among the samples within each treatment group (Figure 2). As expected, large differences were noted between the expression patterns of untreated chondrocytes and those receiving IL-1β alone, illustrating the dramatic impact of IL-1β stimulation on chondrocyte biology. In stark contrast, however, the transcription profiles of the treatment groups cultured in the presence of glucosamine, both with and without IL-1β, clustered closely together on one branch of the hierarchical tree with high correlation. The striking similarity of expression patterns between these two groups indicated that, in the presence of glucosamine, IL-1β had little influence on global transcription patterns in chondrocytes. Furthermore, the expression patterns of the treatment groups receiving glucosamine, both with and without IL-1β, together shared a greater degree of similarity with the samples in the untreated control group than the group receiving IL-1β alone. The results of the individual treatment groups are now discussed in more detail.

### Effects of glucosamine alone on chondrocyte global expression patterns

Overall, relative to untreated controls, incubation of chondrocytes with glucosamine alone led to a global shift in expression across the genome. Of the 2,433 genes that showed a significant response (*P *< 0.0001), expression of 1,506 genes decreased while expression of 927 genes increased. A list of genes with known function that showed the greatest response (a decrease in RNA signal by at least 80%, or an increase of at least fivefold) is presented in Table 2.

No clear pattern was observed among the types of genes that showed the greatest increase in RNA levels following exposure to glucosamine. Curiously, MMP-13 (also termed collagenase-3) – a protease specific for type II collagen, a primary ECM component of articular cartilage – was among the few genes whose expression was strongly stimulated (in this case approximately eightfold) by glucosamine. Interestingly, several of the genes that showed a strong reduction in expression participate in regulation of the cell cycle and cell division.

The data shown in Tables 3 and 4 further describe the effects of glucosamine alone on chondrocyte expression patterns. Of relevance to OA, incubation with exogenous glucosamine alone led to about a twofold reduction in the expression of several genes associated with the synthesis of cartilage ECM, such as collagen type II, biglycan, and cartilage link protein, as well as a twofold increase in MMP-3 RNA (Table 4). Beyond these, glucosamine had no significant stimulatory effect at any level on the expression of genes associated with the synthesis and maintenance of articular cartilage ECM. These include articular cartilage collagens, types VI, IX, XI and X, as well as aggrecan. No significant increase in the synthesis of genes important for glycosaminoglycan synthesis was observed, including UDP-glucose pyrophosphorylase, UDP-glucose dehydrogenase and hyaluronan synthase, among others (data not shown).

### Effects of IL-1β alone on global expression patterns in chondrocytes

In our assays, IL-1β alone significantly affected the expression of 2,813 genes (*P *< 0.0001). Among these, 1,675 genes showed a reduction in RNA level while 1,138 genes showed increased expression. A list of genes with known function that showed the greatest response to IL-1β (a decrease by at least 80% or an increase of at least fivefold) is presented in Table 5. As seen from the table, incubation with IL-1β dramatically increased the expression of numerous inflammatory cytokines (IL-1α, IL-1β, IL-6, and IL-23), chemokines (CCL3, CCL5, CCL7, CXCL1, CXCL2, and CXCL5), and growth factors (BMP-2, BMP-6, BMP-7, and FGF-9) as well as proteins involved in the synthesis of prostaglandin E_2 _and NO (phospholipase A_2_, COX-2, prostaglandin E_2 _synthase, and NO synthase). By increasing the expression of matrix metalloproteinases (MMP-3, MMP-9, MMP-10, MMP-12, and MMP-13) while inhibiting the expression of genes encoding essential components of the ECM (such as collagen type II and aggrecan-1), the elevated IL-1β also shifted the biology of the chondrocytes, at least at the RNA level, toward articular cartilage degradation. The response of the chondrocytes to stimulation with IL-1β alone was therefore largely consistent with previous single-gene studies [3,27] and high-throughput studies, and further demonstrated the potency of this cytokine as a mediator of inflammation and its capacity to influence chondrocyte metabolism, particularly with respect to arthritis.

### Effects of IL-1 on expression patterns of chondrocytes cultured in the presence of glucosamine

Although glucosamine alone had no direct stimulatory effect on the expression of genes associated with ECM synthesis, as indicated in Tables 3, 4, 5 it proved to be a surprisingly potent, broad-spectrum inhibitor of IL-1 stimulation across the entire genome. Indeed, of the 2,813 genes whose expression was significantly affected by IL-1 alone, either increased or decreased, 6-hour preincubation of the chondrocytes with glucosamine significantly blocked that effect in 2,055 genes (~73%). Furthermore, of the IL-1β-sensitive genes whose altered expression was not inhibited by exogenous glucosamine, closer examination of the data revealed that glucosamine alone had the same type of effect as IL-1 on that gene and, likewise, enhanced or repressed expression (see Tables 3, 4, 5).

With regard to arthritis, Tables 3 and 4 represent genes with important roles in inflammation and articular cartilage ECM maintenance that were significantly affected by IL-1 alone. In parallel, the response of these genes to glucosamine alone, and to IL-1β in the presence of glucosamine, is also shown. As reflected in these tables, glucosamine significantly inhibited the expression of the majority of genes whose products are responsible for driving the arthritogenic activities of IL-1. These products include the primary cytokines, chemokines, synthetic and proteolytic proteins associated with the pathology of OA. The protection, however, was not complete or uniform for all genes. For example, the IL-1β-enhanced expression of COX-2, NO synthase, and IL-6 was inhibited by >90%; however, in certain other cases it was less inhibited – such as CD44, which was inhibited by ~50%. Expression of a few key genes, such as collagen type II and MMP-13, appeared not to be protected; however, Tables 3 and 4 show that expression of these genes was also downregulated and enhanced, respectively, by prior incubation with glucosamine alone.

The protective effect of glucosamine was not limited specifically to inflammatory genes and ECM-related genes, but encompassed numerous gene types across the entire. Interestingly, the inhibitory effect of glucosamine toward IL-1 signaling appeared far more influential on genes whose expression was enhanced by IL-1β stimulation than on those genes in which expression was repressed. This is best illustrated in Table 5, where glucosamine was found to significantly block IL-1β-induced expression in 107 of the 110 genes whose RNA level was increased greater than fivefold. Expression of two of the exceptions, MMP-13 and ring finger protein 28, was found similarly enhanced by glucosamine alone. Only the IL-1-enhanced expression of the IL-13 receptor α_2 _chain was unaffected (*P *< 0.0001) by glucosamine. Conversely, of the 35 genes whose transcription was repressed >80% by IL-1, preincubation with glucosamine significantly prevented that effect in only 10 genes. Glucosamine alone, however, also downregulated transcription of the remaining 25 genes. With very few exceptions, therefore, preincubation with glucosamine effectively inhibits the response of chondrocytes to subsequent stimulation with IL-1β.

**Figure 3:**
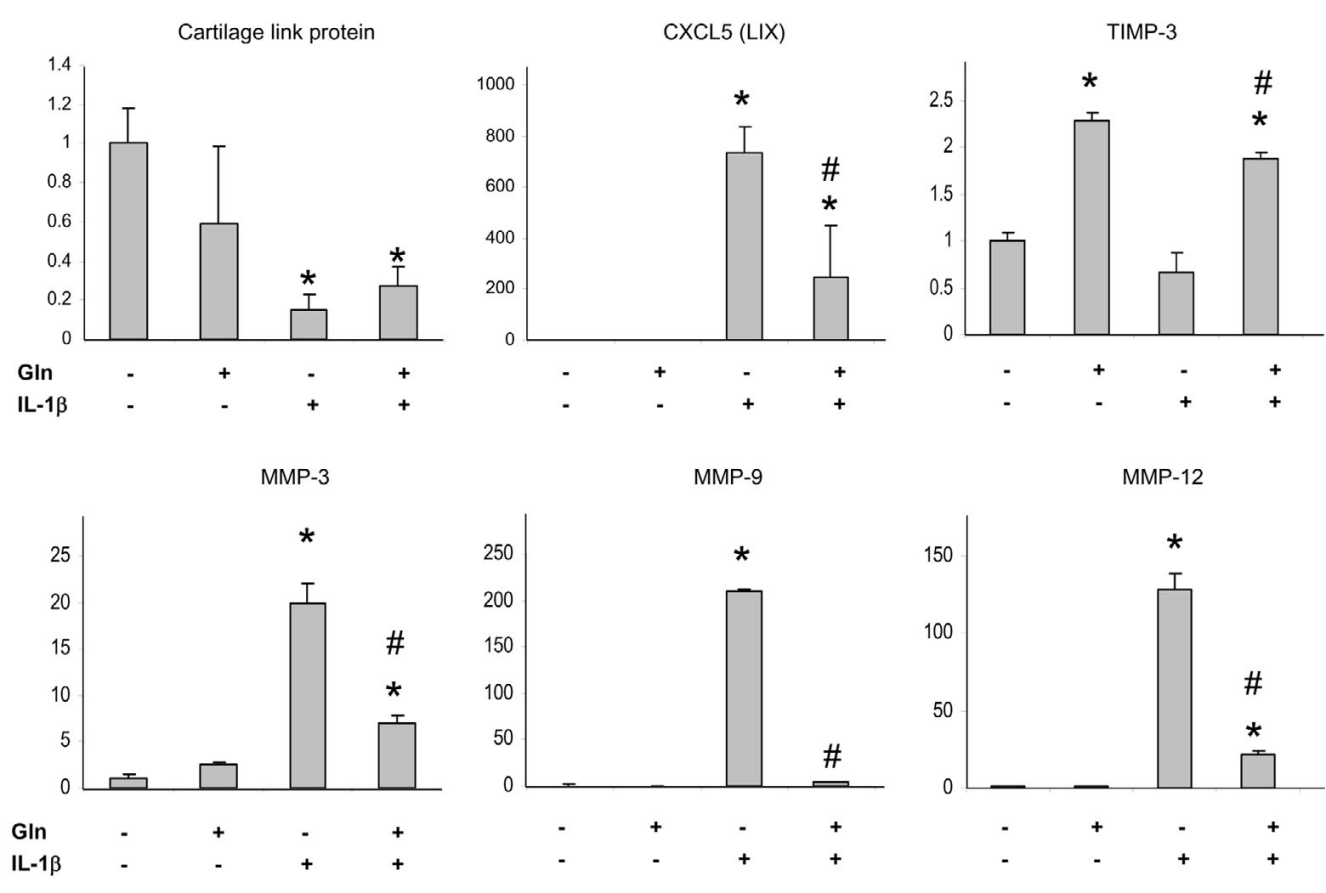
Real-time PCR analyses of cDNA generated from chondrocytes treated with glucosamine (Gln) and IL-1β. Data presented as the mean + standard error of the mean (*n *= 3). *P < 0.05 versus untreated, #*P *< 0.05 versus IL-1β alone.

In an effort to validate the results of our microarray analyses, using total RNA from the individual samples we generated cDNA and used real-time PCR to determine the relative changes for several genes of interest. As shown in Figure 3, the patterns of expression of the six genes analyzed in this manner were very similar to those from the microarray data.

## Discussion

Using global expression analyses we studied the influence of glucosamine on the molecular biology of the chondrocyte, both alone and following challenge with IL-1β. Somewhat contrary to popular belief and several published reports, we found no evidence that elevated levels of exogenous glucosamine increased the transcription of genes with products associated with the synthesis of articular cartilage ECM components. Unlike the dramatic response elicited by IL-1β alone, whereby dozens of genes in related classes were strongly affected, many by more than 100-fold, the response to glucosamine was much more subtle. Since only a handful of genes were stimulated more than fivefold, we were not able to assemble a clear image of the net effect of glucosamine alone on chondrocyte biology. It was only in samples challenged with IL-1β that a beneficial effect of glucosamine became evident. Indeed, preincubation with glucosamine rendered the chondrocytes essentially unresponsive to subsequent IL-1 stimulation, and thereby proved to be highly chondroprotective. Our findings here are in close agreement with previous single-gene analyses that showed elevated glucosamine inhibited the IL-1-induced expression of isolated genes such as COX-2, NO synthase and IL-6, among others [13,17-20,22]. It is only through the use of microarray technology, as shown here – which permits simultaneous examination of the relative expression of all known genes – that a comprehensive profile can be developed and the breadth of glucosamine-mediated chondroprotection is fully appreciated.

It should be emphasized that the conditions used in this study represent supraphysiological levels of both glucosamine and IL-1. As such, the results are not reflective of the *in vivo *situation encountered following oral administration of glucosamine in OA; nor are they representative of the amplitude of the biological response that may be achieved. This, however, was not the intention behind the experimental design. Our goal was to provide a comprehensive depiction of the effects of glucosamine on the biology of the chondrocyte. We therefore selected doses of both IL-1 and glucosamine that provided robust responses in our bioassay (NO synthesis). When attempting to interpret the ensuing microarray data, therefore, we could be confident that the cells received functional doses of both molecules. In doing this, we aimed to provide a representation of the types of effects that might be achieved if glucosamine could be delivered to the joint tissues at functionally effective doses.

Although the underlying mechanisms that drive OA are not completely understood, a broader appreciation for the involvement of inflammatory cytokines such as IL-1 has emerged over the past several years [28]. Our results here underscore the potential of IL-1β as an arthritic mediator with the capacity to drive the key pathways typically associated with the pathogenesis of OA. As shown here, as well as by others, IL-1 abruptly shifts the metabolism of the chondrocyte stimulating the expression of numerous genes, such that the cells responsible for maintenance of the articular cartilage matrix are converted into effector cells that degrade the matrix and produce numerous inflammatory and chemoattractant molecules. Although our experiments were performed with high concentrations of IL-1 that far exceed physiological levels, IL-1 is a potent cytokine with a strong spare receptor effect. It is easy to envision how persistent exposure at much lower levels may more slightly, but fundamentally, alter the biology of the chondrocytes, effecting over time a gradual but steady shift toward cartilage degradation. Pharmacologics that can effectively inhibit the activities of this and other inflammatory cytokines could therefore be highly beneficial in the treatment of arthritic conditions.

Acetaminophen and nonsteroidal anti-inflammatory drugs such as ibuprofen and naproxen (and until recently celecoxib and rofecoxib) are the most widely used pharmacologics in the management of OA [29]. The latter are thought to specifically inhibit the activity of the cyclooxygenases, enzymes that mediate the conversion of arachidonic acid to prostaglandins. As demonstrated here, however, prostaglandin synthesis only accounts for one of many of the pathologic processes that occur in response to inflammatory cytokines such as IL-1. Thus, while cyclooxygenase inhibitors are useful in the management of certain symptoms, primarily pain, much of the underlying pathogenesis of OA goes unchecked. In our experiments exogenous glucosamine effectively rendered the chondrocytes unresponsive to IL-1 stimulation. A potential advantage of this amino sugar is therefore its capacity to inhibit inflammatory signaling across the entire spectrum.

In previous work, we and others have demonstrated that IL-1-mediated NF-κB activation and nuclear translocation were reduced in chondrocytes in the presence of elevated exogenous glucosamine [17,22]. We recently found that glucosamine also inhibits aspects of inflammatory signaling by TNFα (unpublished observation). Others have shown its ability to block the effects of lipopolysaccharide in chondrocytes as well as other cell types. Lipopolysaccharide and IL-1 have overlapping cell signaling pathways mediated through Toll-like and IL-1 receptors, respectively [30,31]. Ligand binding of these receptors leads to activation of Myd88, IL-1 receptor-activated kinases, and TNF receptor-associated factor 6, which in turn activates cytosolic NF-κB. Inflammatory TNF receptor 1 signaling, mediated through the TNF receptor-associated death domain adaptor protein, and interaction with receptor interacting protein and TNF receptor-associated factor 2 also work to activate NF-κB. For these inflammatory agents, therefore, NF-κB activation appears perhaps the earliest common site for intervention by glucosamine. Whether this inhibition occurs through direct interaction with glucosamine or downstream products of the hexosamine pathway, or is a secondary consequence of other cellular processes influenced by elevated glucosamine, has yet to be established. While the NF-κB pathway is a central player in inflammatory signal transduction, IL-1 and TNF also share the capacity to activate the stress-activated protein kinase/c-Jun N-terminal kinase and p38 mitogen-activated protein kinase, as well as others [32,33]. In previous work, however, glucosamine did not appear to inhibit nuclear translocation of activator protein 1 following stimulation of chondrocytes with IL-1 [22]; however, the relationship between this signaling pathway and glucosamine has not been studied in detail. Given the breadth of chondroprotection provided by glucosamine as shown here, it is likely that these signaling pathways also are functionally blocked by glucosamine.

In light of the capacity of glucosamine to influence signal transduction and cellular metabolism, an additional consideration is how sustained exposure to elevated levels may influence chondrocyte biology, and in turn influence the vitality of articular cartilage in the long term. From our experiments, it appears that glucosamine alters the overall responsiveness of the chondrocyte to inflammatory signaling. Along these lines, elevated glucosamine has also been found to cause a loss of sensitivity to stimulation of insulin and IGF-1 receptor tyrosine kinase activity in certain cells in culture, and leads to insulin resistance in experimental animals [34-36]. How consumption of glucosamine may alter the capacity of the chondrocyte to respond to other external stimuli, including various anabolic signals, therefore remains uncertain. These studies have not been undertaken but should be considered in light of our results here and of the popularity of glucosamine as a nutritional supplement.

Despite the results of our microarray and other *in vitro *assays that have demonstrated the capacity of glucosamine to impede inflammatory stimulation *in vitro*, the clinical value of glucosamine in the treatment of OA remains controversial [11,37]. The recently published results of the Glucosamine/chondroitin Arthritis Intervention Trial showed that, across the larger population of patients with OA, glucosamine and chondroitin sulfate were no more effective than placebo [12]. In a predetermined subpopulation of those with moderate to severe pain, however, there appeared to be significant benefit. The basis for these discrepancies is unknown. One possible explanation may be the relative participation of inflammatory cytokines in different subpopulations; perhaps the effects of glucosamine and chondroitin are better realized in patients with more severe OA that have greater involvement of IL-1. Another reason for the limited clinical response overall may be the extent to which glucosamine enters the human circulation and the joint space after the recommended oral dose. Several studies suggest that effective intra-articular concentrations may not always be achieved [38]. The route of administration may therefore be key to reach the necessary concentration of glucosamine to take full advantage of its potential effects. A method by which it may be possible to generate effective levels of glucosamine in the joint tissues is through local gene transfer of the enzyme glutamine 6-phosphate-amido-transferase, which is a limiting enzyme in glucosamine synthesis. The proof of concept has already been demonstrated [39,40], laying the foundation for new directions to exploit the therapeutic potential of glucosamine in OA.

## Conclusion

Using the assays used in the present study, the anti-arthritic properties attributed to the consumption of glucosamine do not appear related to cartilage matrix synthesis, but more related to its ability to globally inhibit the deleterious effect of IL-1β signaling. The data suggest that the potential benefit to ingestion or administration of glucosamine lies primarily with its anti-inflammatory properties and not with the replenishment of the ECM. These results support the use of glucosamine as an anti-arthritic agent if it can be administered at the appropriate dosage to joint tissues.

## Abbreviations

DMEM = Dulbecco's modified Eagle's medium; ECM = extracellular matrix; IL = interleukin; NF-κB = nuclear factor kappa B; NO = nitric oxide; OA = osteoarthritis; PCR = polymerase chain reaction; TNF = tumor necrosis factor.

## Competing interests

The authors declare that they have no competing interests.

## Authors' contributions

J-NG conceived of and carried out the study. EG and SCG participated in the study design and coordination. MPP performed the array hybridization and the data analysis. MLB, EAD, JDK, PPL, KRP, JSS and RSW contributed to scientific discussions and helped to draft the manuscript. All authors read and approved the final manuscript.

## Figures and Tables

**Table 1:** Primer sequences used to quantify gene expression with real-time PCR

Gene	Sequence (5' to 3')
Cartilage link protein	Forward, GCATCAAGTGGACCAAGCTA
	Reverse, GTAACTCCAATGCCACCACA
Collagen alpha 1 type II	Forward, GTGGAGCAGCAAGAGCAAGGA
	Reverse, CTTGCCCCACTTACCAGTGTG
CXCL5 (LIX)	Forward, CACCCTGCTGGCATTTCTG
	Reverse, AACCATGGCCGAGAAAGGA
MMP-3	Forward, CTGGAATGGTCTTGGCTCAT
	Reverse, CTGACTGCATCGAAGGACAA
MMP-9	Forward, CCACCGAGCTATCCACTCAT
	Reverse, GTCCGGTTTCAGCATGTTTT
MMP-12	Forward, TGCAGCTGTCTTTGATCCAC
	Reverse, TCCAATTGGTAGGCTCCTTG
TIMP-3	Forward, GTACACAGGGCTGTGCAACTTTGTG
	Reverse, CTTCTGCCGGATGCAGGCGTAGTG
EF1alpha	Forward, GATGGCCCCAAATTCTTGAAG
	Reverse, GGACCATGTCAACAATGGCAG

**Table 2:** Genes whose expression showed the greatest change following incubation of chondrocytes with elevated glucosamine alone

**Genes whose mean RNA levels were reduced >80% relative to untreated control cultures**			
Anaphase-promoting complex subunit 8	Cyclin-dependent kinase inhibitor 3	Kinesin-like protein 1	Selenium binding protein 2
Bone morphogenetic protein 4	Cytoskeleton associated protein 2	Kinesin-related protein KRP1	Shc SH2-domain binding protein 1
Calmodulin	DVS27-related protein	Lactose operon repressor^a^	Solute carrier family 4, member 4
Carbonic anhydrase 3	Dynein, cytoplasmic, intermediate chain 1	Microtubule-associated motor KIF4	Sphingomyelin phosphodiesterase 3, neutral
Cell cycle protein division p55CDC	ER transmembrane protein Dri 42	NAD-dependent 15-hydroxyprostaglandin deshydrogenase	Testin (TES1/TES2)
Cell division cycle 2 homolog A	Esk splice form 1	Neural precursor cell expressed, developmentally downregulated gene 4A^a^	Thymidine kinase 1
Cell proliferation antigen Ki-67	Frizzled related protein (sfrp2 gene)^a^	Neuropilin	Topoisomerase (DNA)2 alpha
Cell-cycle-dependent 350K nuclear protein	G2/mitotic-specific cyclin B_1_^a^	NUF2R protein	Transforming acidic coiled-coil containing protein 3
c-*fos*-induced growth factor (vascular endothelial growth factor D)	Glycine amidinotransferase (l-arginine:glycine amidinotransferase)	Pituitary tumor-transforming 1	Ubiquitin conjugating enzyme
Chemokine (C–X–C motif) ligand 12^a^	Heat shock protein 90 beta	Polo-like kinase homolog	Vascular endothelial growth factor D precursor
Clathrin light chain A (Lca)	Hyaluronon mediated motility receptor (RHAMM)	Protein regulating cytokinesis 1	
Cyclin B_1_	Insulin-like growth factor binding protein 3	Rac GTPase-activating protein 1^a^	

**Genes whose mean RNA levels were increased more than fivefold relative to untreated controls**			

Aldose reductase-like protein	High mobility group AT-hook 1	Plasminogen activator inhibitor 2 type a	Vesicle-associated membrane protein 1
ATP-binding cassette, subfamily G (WHITE), member 1	Matrix metalloproteinase 13	Smhs 1 protein	V-maf musculoapaneurotic fibrosarcoma oncogene family protein B
CD28 antigen	Myo-inositol 1-phosphate synthase A1	Sodium-coupled ascorbic acid transporter 2	
FXYD domain containing ion transport regulator 2	NADH-ubiquitone oxidoreductase MLRQ subunit	Solute carrier family 1, member 3	

^a^Multiple probe sets.

**Table 3:** Relative signal values of inflammatory genes significantly affected by IL-1 and/or glucosamine

Gene	Treatment group			
	No glucosamine, no IL-1	Glucosamine, no IL-1	IL-1, no glucosamine	Glucosamine with IL-1
Arginosuccinate synthetase	6.3	19.4	2247.3	267.2
β-Nerve growth factor	16.1	21.6	196.2	13.0
Bone morphogenetic protein 2	42.7	57.7	375.4	100.2
Bone morphogenetic protein 4	494.9	77.3	147.7	68.2
Bone morphogenetic protein 6	60.9	13.9	435.9	85.9
Bone morphogenetic protein 7	0.9	5.8	76.2	8.2
CCL3 (MIP-1a)	-	0.1	690.7	2.6
CCL5 (RANTES)	2.1	2.7	310.1	8.1
CCL7 (MCP-3)	32.2	69.4	3076.0	662.8
CCL22 (MDC)	0.3	0.6	47.2	5.4
Cdc42 GTPase inhibiting protein	105.0	116.5	189.4	124.4
Colony-stimulating factor 1	126.9	51.1	307.8	140.8
Colony-stimulating factor 2	-	20.1	1210.7	17.6
Colony-stimulating factor 3	-	6.0	1253.7	6.5
CXCL1 (GROa)	168.2	36.6	1730.6	496.2
CXCL2 (GROb)	-	37.5	2988.2	92.4
CXCL5 (LIX)	2.0	2.4	441.3	16.2
CXCL10 (IP-10)	10.7	8.1	74.4	15.6
CXCL11 (I-TAC)	-	-	110.2	3.4
CXCL12 (SDF-a/b)	122.8	5.9	88.7	10.9
Cyclooxygenase 1	184.0	593.4	192.2	493.1
Cyclooxygenase 2	61.9	117.0	3493.9	405.5
Cysteine knot superfamily 1 (bone morphogenetic protein antagonist 1)	460.4	308.6	2174.9	732.7
Cytokine-induced neutrophil chemoattractant-2	15.2	54.4	3466.1	624.2
Dual-specificity phosphatase 6	179.9	88.9	658.7	109.9
Endothelial PAS domain protein 1	69.9	38.0	208.9	52.2
Fibroblast growth factor 7	1294.4	1078.9	1318.9	1745.9
Fibroblast growth factor 9	12.0	29.5	155.7	26.7
Fibroblast growth factor receptor 1	605.2	653.7	329.3	638.5
I-κBα	126.9	81.7	1108.4	399.0
I-κBβ	55.6	96.6	298.2	139.2
I-κBγ (NF-κB p105 subunit)	124.2	107.9	471.2	163.6
Insulin-like growth factor binding protein 3	81.6	7.4	249.9	10.4
Insulin-like growth factor binding protein 5	174.4	569.0	737.8	229.5
IL-1α	1.5	3.2	175.3	4.5
IL-1β	-	2.4	119.4	2.7
IL-6	57.3	53.4	1717.8	159.0
IL-11	-	-	124.4	-
IL-13 receptor, α 1	174.8	144.5	337.4	148.0
IL-23α subunit p19	-	-	766.5	-
Janus kinase 2	89.7	102.9	251.0	128.7
JunB proto oncogene	129.5	89.0	377.8	148.4
Mitogen-activated protein kinase phosphatase (CPG21)	19.1	47.5	300.2	52.3
Nitric oxide synthase 2	0.1	2.1	1033.9	66.1
Ornithine aminotransferase	1080.5	1125.0	868.6	1059.1
Ornithine decarboxylase antizyme inhibitor	322.1	239.4	336.9	186.6
Phospholipase A_2_, group 2A	175.5	44.7	2085.9	358.0
Phospholipase A_2_, group 4A	269.9	303.2	668.2	339.7
Phospholipase A_2_, group 5	44.0	18.2	260.7	66.7
Platelet-derived growth factor A	439.8	492.9	745.9	597.5
Prostaglandin E_2 _synthase	32.8	46.9	3845.8	183.7
Pyrroline-5-carboxylate	602.5	643.8	348.3	609.3
Rel-B	31.6	28.7	186.2	71.5
Small inducible cytokine A_2_	221.4	711.0	4835.8	2945.5
Small inducible cytokine A_20_	26.9	137.8	4206.1	1609.7
Solute carrier family 7 (cationic amino acid transporter), member 8	8.1	19.4	560.3	21.7
Sox-4	533.0	460.8	248.5	572.2
TNF-induced protein 6	119.8	49.3	262.3	71.1

**Table 4:** Relative signal values of articular cartilage extracellular-matrix-related genes significantly affected by IL-1 and/or glucosamine

Gene	Treatment group			
	No glucosamine, no IL-1	Glucosamine, no IL-1	IL-1, no glucosamine	Glucosamine with IL-1
A disintegrin and metalloproteinase domain 15	142.9	121.3	64.9	98.3
A disintegrin and metalloproteinase domain 17	434.6	533.1	897.4	616.6
ADAMTS-1	457.0	202.1	996.0	359.7
ADAMTS-4	79.2	149.8	76.0	149.4
Aggrecan-1	162.6	154.7	47.1	117.3
Biglycan	80.9	26.1	126.8	19.2
Cartilage link protein	662.1	284.3	75.9	166.7
Chondroitin sulfate proteoglycan 2 (Versican)	135.3	45.3	418.1	60.0
Collagen type II alpha 1, chain precursor	106.4	42.0	3.8	20.6
Collagen type III alpha 1	2710.2	2720.0	1320.4	2242.6
Collagen type VIII alpha 1	622.6	362.5	857.9	493.8
Collagen type XI alpha 1	2510.6	2090.9	1471.5	1830.9
Laminin α-4 chain precursor	238.3	510.2	158.6	410.8
Matrix metalloproteinase 2	1014.0	1388.4	1825.7	1886.2
Matrix metalloproteinase 3	894.7	1966.4	5787.4	4223.6
Matrix metalloproteinase 9	-	11.1	2194.0	36.5
Matrix metalloproteinase 10	-	7.8	271.2	39.6
Matrix metalloproteinase 12	4.9	13.4	1132.0	84.9
Matrix metalloproteinase 13	268.3	2126.9	4497.4	3871.2
Syndecan 2	1515.8	1292.9	656.8	963.4
Syndecan 4	927.7	796.8	2049.2	1327.5
Thrombospondin 4 precursor	2535.4	2217.7	1224.9	1662.6
TIMP-1	1786.8	1857.2	3049.8	2311.4
TIMP-2	995.9	937.0	726.9	923.1
TIMP-3	1317.1	1983.9	1105.3	1859.0

**Table 5:** Genes whose expression showed the greatest change following incubation of chondrocytes with elevated IL-1β alone: genes of known function whose expression was most affected by IL-1 alone

**Genes whose RNA levels were reduced >80% relative to untreated control cultures**			
• A disintegrin and metaloproteinase domain 33	• c-*fos*-induced growth factor (vascular endothelial growth factor D)	Kruppel associated box zinc finger 1	• Smoothelin
• Actin alpha 1	• Collagen type II alpha 1, chain precursor	• Microfibril-associated glycoprotein precursor	Solute carrier family 39 (iron-regulated transporter), member 1
Aggrecan 1	Crystallin, alpha B	• Midline 1	• Sphingomyelin phosphodiesterase 3, neutral
• Ankyrin-like repeat protein	Distal-less homeobox	• Mitochondrial ribosomal protein L53	• Transforming growth factor, beta 2
• Annexin III (Lipocortin III)	• DVS27-related protein	Myocilin precursor	• Zinc finger protein SLUG (neutral crest transcription factor Slug)
Cadherin-8 precursor	• Dynein, cytoplasmic, intermediate chain 1	• NAD-dependent 15-hydroxyprostaglandin dehydrogenase	
Calpain 6	• Enolase 3, beta	Osteomodulin (osteoadherin)	
• Carbonic anhydrase 3	Four and a half LIM domains 1	• Palmdelphin^a^	
Cartilage link protein 1 ^a^	• Heat shock protein HSP 90 beta^a^	• Phosphoribosyl pyrophosphate synthestase 2	
• Caveolin 3	Insulin-like growth factor binding protein 4 precursor	• Programmed cell death protein 7	

**Genes whose RNA levels were increased more than fivefold relative to untreated controls**			

Adaptor protein with pleckstrin homology and src homology 2 domains	Cyclooxygenase-2	Keratin, type II cryosqueletal 8	Plasminogen activator, urokinase receptor
Adenosine A2B receptor	Cytochrome P450, family 26, subfamily b, polypeptide 1	Lactose operon repressor	Prostaglandin E synthase ^a^
Adenosine monophosphate deaminase 3	Cytochrome P450, subfamily 7B, polypeptide 1^a^	Laminin beta-2 chain precursor	Purigenic receptor P2Y, G-protein coupled 2
Aldose reductase-like protein	Cytokine-induced neutrophil chemoattractant-2^a^	MAP-kinase phosphatase (cpg21)	RAB27B, member RAS oncogene family
Apoptotic death agonist BID	EGL nine homolog 3 (C. elegans)	Matrix metalloproteinase 3	Rat VL30 element ^a^
Arginosuccinate synthetase	Endothelial cell-specific molecule 1	Matrix metalloproteinase 9^a^	Receptor-interacting serine-threonine kinase 2
ATP-binding cassette, sub-family G (WHITE), member 1	Epiregulin precursor	Matrix metalloproteinase 10	RelB
BCL2-related protein A1	Fatty acid binding protein 4	Matrix metalloproteinase 12	Retinol-binding protein 1
Beta-nerve growth factor	F-box protein Fbx5	• Matrix metalloproteinase 13	• Ring finger protein 28
Bloom's syndrome protein homolog (mBLM)	Fibroblast growth factor 9	Mesothelin	Schlafen 4
Bone morphogenetic protein 2^a^	Follistatin	Microtubule-associated protein	Small inducible cytokine A2
Bone morphogenetic protein 6^a^	Fos-like antigen 1	6 ^a^	Smhs1 protein
Bone morphogenetic protein 7	Gardner-Rasheed feline sarcoma viral (Fgr) oncogen homolog	Mitochondrial solute carrier protein	Solute carrier family 1, member 1
Brain-specific angiogenesis inhibitor 1-associated protein 2	GATA-binding protein 2	Myotubularin related protein 7	Solute carrier family 1, member 3
CCL3 (Mip-1a)	Gro	NADH-ubiquinone oxidoreductase MLRQ subunit	Solute carrier family 7 (cationic amino acid transporter, y^+ ^system), member 1
CCL5 (RANTES)	Growth arrest specific 7	Neurofilament, heavy polypeptide	Solute carrier family 11, member 2
CCL7 (MCP-3)	GTP cyclohydrolase 1	Neuron specific protein PEP-19 (Purkinje cell protein 4)	Solute carrier family 20 (phosphate transporter), member 1
CD44 antigen	High mobility group AT-hook 1	Neuropeptide Y	Superoxide dismutase 2
Chemokine receptor (LCR1)^a^	IκB-α	Neurospin precursor	T-cell death associated gene
Cholesterol 15-hydroxylase	IκB-β (nuclear factor kappa B p105 subunit)	Nitric oxide synthase 2	Testis-specific protein Bs13
Claudin-3	Inhibin beta-A	Organic cation transporter OCTN1	Tissue factor pathway inhibitor 2
Colony stimulating factor 2 (granulocyte-macrophage)	Inhibitor of apoptosis protein 1	Parvalbumin	Tissue-type transglutaminase
Colony stimulating factor 3	Insulin-like growth factor-binding protein 5	Phosphodiesterase 4B, cAMP-specific (dunce (Drosophila) homolog phosphodiesterase E_4_)	Toll-like receptor 2
Complement factor B precursor (C3/C5 convertase)	Interleukin 1 alpha	Phospholipase A2, group 5	Tumor necrosis factor alpha-induced protein 2^a^
CXCL1 (GROa)	Interleukin 1 beta	Phospholipase A2, group IIA (platelets, synovial fluid)	Tumor necrosis factor receptor superfamily member 5 precursor
CXCL2 (GROb)	Interleukin 6 (interferon, beta 2)	Phospholipid scramblase 1	Tumor necrosis factor receptor superfamily member 9 precursor
CXCL5 (LIX)	Interleukin 23, alpha subunit p19	Plasminogen activator inhibitor 2 type A	Uridine phosphorylase
CXCL10 (IP-10)	• Interleukin 13 receptor, alpha 2	Plasminogen activator, tissue	

^a^Represents multiple probe sets. Bold circles indicate genes for which the IL-1-mediated effects on transcription were not inhibited by exogenous glucosamine (see text).
